# The Surgical Management of Brain Metastases in Non-Small Cell Lung Cancer (NSCLC): Identification of the Early Laboratory and Clinical Determinants of Survival

**DOI:** 10.3390/jcm10174013

**Published:** 2021-09-05

**Authors:** Matthias Schneider, Niklas Schäfer, Christian Bode, Lars Eichhorn, Frank A. Giordano, Erdem Güresir, Muriel Heimann, Yon-Dschun Ko, Jennifer Landsberg, Felix Lehmann, Alexander Radbruch, Christina Schaub, Katjana S. Schwab, Johannes Weller, Ulrich Herrlinger, Hartmut Vatter, Patrick Schuss

**Affiliations:** 1 Center of Integrated Oncology (CIO) Bonn, Department of Neurosurgery, University Hospital Bonn, 53127 Bonn, Germany; erdem.gueresir@ukbonn.de (E.G.); muriel.heimann@ukbonn.de (M.H.); hartmut.vatter@ukbonn.de (H.V.); patrick.schuss@ukbonn.de (P.S.); 2 Center of Integrated Oncology (CIO) Bonn, Department of Neurology, Division of Clinical Neuro-Oncology, University Hospital Bonn, 53127 Bonn, Germany; niklas.schaefer@ukbonn.de (N.S.); christina.schaub@ukbonn.de (C.S.); johannes.weller@ukbonn.de (J.W.); ulrich.herrlinger@ukbonn.de (U.H.); 3 Department of Anesthesiology and Intensive Care, University Hospital Bonn, 53127 Bonn, Germany; christian.bode@ukbonn.de (C.B.); lars.eichhorn@ukbonn.de (L.E.); felix.lehmann@ukbonn.de (F.L.); 4 Center of Integrated Oncology (CIO) Bonn, Department of Radiation Oncology, University Hospital Bonn, 53127 Bonn, Germany; frank.giordano@ukbonn.de; 5 Center of Integrated Oncology (CIO) Bonn, Department of Oncology and Hematology, Johanniter Hospital Bonn, 53113 Bonn, Germany; yon-dschun.ko@bn.johanniter-kliniken.de; 6 Center of Integrated Oncology (CIO) Bonn, Department of Dermatology and Allergy, University Hospital Bonn, 53127 Bonn, Germany; jennifer.landsberg@ukbonn.de; 7 Center of Integrated Oncology (CIO) Bonn, Department of Neuroradiology, University Hospital Bonn, 53127 Bonn, Germany; alexander.radbruch@ukbonn.de; 8 Center of Integrated Oncology (CIO) Bonn, Department of Internal Medicine III, University Hospital Bonn, 53127 Bonn, Germany; katjana.schwab@ukbonn.de

**Keywords:** surgical management, brain metastases, lung cancer, overall survival

## Abstract

Background: Brain metastases (BM) indicate advanced states of cancer disease and cranial surgery represents a common treatment modality. In the present study, we aimed to identify the risk factors for a reduced survival in patients receiving a surgical treatment of BM derived from non-small cell lung cancer (NSCLC). Methods: A total of 154 patients with NSCLC that had been surgically treated for BM at the authors’ institution between 2013 and 2018 were included for a further analysis. A multivariate analysis was performed to identify the predictors of a poor overall survival (OS). Results: The median overall survival (mOS) was 11 months (95% CI 8.2–13.8). An age > 65 years, the infratentorial location of BM, elevated preoperative C-reactive protein levels, a perioperative red blood cell transfusion, postoperative prolonged mechanical ventilation (>48 h) and the occurrence of postoperative adverse events were identified as independent factors of a poor OS. Conclusions: The present study identified several predictors for a worsened OS in patients that underwent surgery for BM of NSCLC. These findings might guide a better risk/benefit assessment in the course of metastatic NSCLC therapy and might help to more sufficiently cope with the challenges of cancer therapy in these advanced stages of disease.

## 1. Introduction

The diagnosis of brain metastases (BM) is associated with a rather poor prognosis, indicating an uncontrolled primary disease that has spread to the otherwise relatively unattainable central nervous system [1]. As for the underlying disease, the most common primary site of malignancy of BM is the lung (20–40%) [2]. In the past, the diagnosis of BM was considered to be an event indicating the terminal stage of an apparently uncontrollable underlying disease and thus further treatment options were considered to be limited [1,3,4]. However, the incidence of BM has increased in recent years due to improved imaging techniques and also due to enhanced survival rates of primary tumors [5]. Progress in the treatment of extracranial disease has led to a more aggressive approach also in the treatment of BM [6,7]. Although there is still no curative treatment, the main purpose of the interdisciplinary treatment of BM is to achieve prolonged, high-quality survival [8,9]. Neurosurgical management has to contribute to this goal with improved preoperative patient selection, intraoperative techniques and monitoring as well as postoperative complication management and patient guidance [10].

Therefore, the aim of this study was to assess the overall survival (OS) rates in patients following surgery for BM from non-small cell lung cancer (NSCLC) as well as to identify preoperatively and early postoperatively collectable factors for a worsened survival that might enable an individual treatment benefit at the earliest possible stage and assist in the management of future surgical cases.

## 2. Materials and Methods

### 2.1. Patients

The records of patients with BM originating from NSCLC who had undergone surgical treatment at the authors’ facility between 2013 and 2018 were screened and relevant data were extracted into a database (SPSS, version 25, IBM Corp., Armonk, NY, USA). The study was conducted in accordance with the Declaration of Helsinki and the protocol was approved by the Ethics Committee of the University Hospital Bonn (Project identification code 250/19). Only patients with histopathologically-proven BM originating from NSCLC were included for a further analysis.

Individual treatment decisions were made at the initial presentation of the patient and during follow-up by the weekly institutional interdisciplinary tumor board meetings for the Central Nervous System, as described previously [11]. 

The factors of interest were demographical and the clinical baseline, radiological features, preoperative laboratory values and functional neurological status at admission were collected and further analyzed. The Karnofsky performance score (KPS) was used to evaluate patients according to their preoperative neurological functional status. The comorbidity burden at the time of surgery was assessed using the age-adjusted Charlson Comorbidity Index (CCI) and patients were divided into two groups according to the previously reported threshold of 10 [12]. A laboratory analysis of C-reactive protein (CRP) and white blood cells (WBC) was performed within 12 h of admission as part of the routine laboratory testing. The WBC counts (normal range 3.9–10.2 G/L) were divided into two groups, ≤12 G/L and >12 G/L, and CRP (normal range 0–3 mg/L) was dichotomized in ≤10 mg/L and > 10 mg/L to indicate a moderate inflammation, as previously described [13]. Patients with NSCLC and BM were further stratified into two groups according to the classification of the American Society of Anesthesiologists (ASA); patients with a preoperative ASA of 1 or 2 and patients with a preoperative ASA ≥ 3, respectively. Perioperative blood transfusions were determined as any allogeneic transfusion of red blood cells (RBC) during or within 5 days of BM surgery. Postoperative prolonged mechanical ventilation (PMV) was defined as prolonged postoperative invasive ventilation for more than 48 h after the surgical treatment of BM [14]. Postoperative complications were defined as the occurrence of a postoperative adverse event (PAE) within 30 days of surgery with the need for subsequent surgical consequences [15,16].

The overall survival (OS) was measured from the day of BM surgery until death or the last observation. Patients for whom no further follow-up information was available (e.g., due to further treatment at external institutions) were excluded from any further analysis. All parameters were compared in terms of the OS. 

### 2.2. Statistics

The data analysis was performed using the computer software package SPSS (version 25, IBM Corp., Armonk, NY, USA). The categorical and binary variables were analyzed in contingency tables using Fisher’s exact test. The Mann–Whitney U-test was chosen to compare the continuous variables as the data were mostly not normally distributed. The OS was analyzed by the Kaplan–Meier method. The relevant clinical factors were entered into a multivariable Cox proportional risk model to predict the overall survival. Results with *p* < 0.05 were considered to be statistically significant. 

## 3. Results

### 3.1. Patient Characteristics

Between 2013 and 2018, a total of 165 patients with NSCLC were surgically treated for BM at the authors’ neuro-oncological center. Of these, 11 patients were excluded after a careful review of the clinical records due to the lack of follow-up information. Therefore, 154 patients with NSCLC and a surgically treated BM were included in a further analysis. The median age was 63 years (range 40–86 years) (Table 1). At admission, patients presented with a median KPS score of 80 (range 30–100). The median OS (mOS) for patients with a surgically treated BM was 11 months (95% CI 8.2–13.8). The causes of death were a diffuse metastatic spread with an initiated palliative therapy concept in 72% followed by a thoracic local malignant recurrence in 11%, a brain metastatic local recurrence in 6% and other reasons in 11% of the brain metastatic patients with NSCLC (among them were postoperative sepsis (2%), a postoperative massive hemorrhage (2%), cardiopulmonary failure following a pulmonary embolism and a myocardial infarction (2%)). Patient-specific details are presented in Table 1. 

### 3.2. Influence of Clinical Admission Status and Tumor Stage

**Figure 1:**
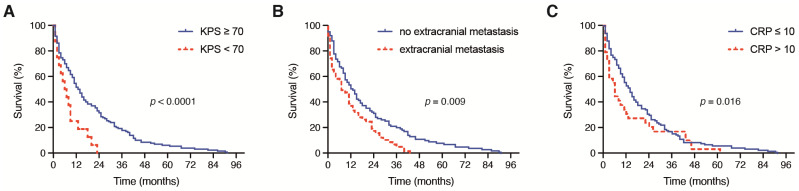
Kaplan–Meier survival curves depicting the influence of (**A**) preoperative KPS (<70, ≥70), (**B**) the presence of further extracranial metastases and (**C**) the preoperative CRP value (≤10 mg/L, >10 mg/L) on the OS in patients with BM derived from NSCLC. BM: brain metastases; CRP: C-reactive protein; NSCLC: non-small cell lung cancer; OS: overall survival.

Of all symptoms initially reported at admission, 83 patients (54%) suffered from manifest/transient neurological deficits alone or in addition to other symptoms, 25 patients (16%) experienced headaches and 16 patients (10%) sustained tumor-related epilepsy. The majority of patients (*n* = 132, 86%) presented with a KPS of ≥70 at admission and 22 patients (14%) had a KPS < 70 before surgery. Patients with a surgically treated brain metastasis of NSCLC and an initial KPS of ≥ 70 achieved a significantly increased OS compared with patients with a preoperative KPS of <70 (*p* < 0.0001). Specifically, the mOS in patients with KPS ≥ 70 was 13 months (95% CI 10.2–15.8) compared with a mOS of 3 months (95% 0.0–6.7) in patients with preoperative KPS < 70 (Figure 1A). 

Patients who were assessed preoperatively with an ASA < 3 had no significant difference in OS compared with patients with a preoperative ASA ≥ 3 (*p* = 0.9). In line with this, the mOS did not significantly differ between patients with an age-adjusted CCI < 10 compared with those with an age-adjusted CCI > 10 (*p* = 0.26).

A total of 57 out of 154 patients with brain metastatic NSCLC (37%) exhibited further extracranial metastases; 97 out of 154 patients with brain metastatic NSCLC (63%) did not exhibit extracranial metastatic lesions. The median OS for patients with further extracranial metastases was 8 months (range 8–43) compared with 14 months (range 0–91) for patients without extracranial metastases (*p* = 0.009) (Figure 1B).

### 3.3. Influence of Preoperative Laboratory Values

Patients with an admission CRP ≤ 10 mg/dL achieved a significantly increased mOS compared with patients with a preoperative CRP > 10 mg/dL (*p* = 0.016). In detail, the mOS in patients with an admission CRP ≤ 10 mg/L was 13 months (95% CI 10.0–16.0) compared with a mOS of 5 months (95% 2.2–7.8) in patients with a preoperative CRP > 10 mg/L (*p* = 0.016, Figure 1B). The OS did not differ significantly between patients with a preoperative WBC ≤ 12 G/L and patients with an admission WBC > 12 G/L (*p* = 0.4).

Patients with a surgically treated BM of NSCLC and an admission CRP > 10 mg/L suffered from a significantly higher mortality rate after 12 months compared with patients with a preoperative CRP ≤ 10 mg/L (73% vs. 49%, *p* = 0.018, OR 2.8). The mortality rate after 12 months did not differ significantly between patients with a preoperative WBC ≤ 12 G/L and WBC > 12 G/L (57% vs. 52%, *p* = 0.5) (Figure 1C).

### 3.4. Influence of Location, Tumor Burden and Perioperative Management

**Figure 2:**
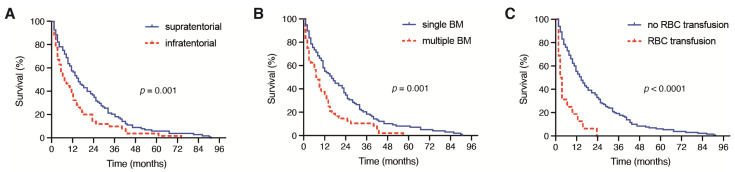
Kaplan–Meier survival curves depicting the influence of (**A**) the presence of a supratentorial or infratentorial localization of BM, (**B**) single versus multiple BM and (**C**) the presence of perioperative RBC on the OS in patients with BM derived from NSCLC. BM: brain metastases; NSCLC: non-small cell lung cancer; RBC: red blood cell; OS: overall survival.

Patients with a supratentorial location of the surgically treated BM achieved a median OS of 14 months (95% CI 10.1–17.9), which significantly differed from the median OS of 6 months (95% CI 2.9–9.1) in patients with an infratentorial location of a surgically treated BM (*p* = 0.001, Figure 2A). 

**Figure 3:**
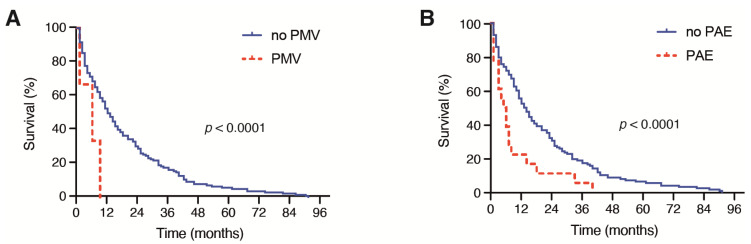
Kaplan–Meier survival curves depicting the influence of (**A**) PMV (>48 h) and (**B**) the development of PAE on the OS in patients with BM derived from NSCLC. BM: brain metastases; CRP: C-reactive protein; NSCLC: non-small cell lung cancer; OS: overall survival; PAE: perioperative adverse events; PMV: prolonged mechanical ventilation.

Patients with multiple BM at the time of surgical treatment achieved a median OS of 7 months (95% CI 2.9–11.1), which was significantly decreased compared with the median OS of 14 months (95% CI 9.1–18.9) in patients with singular BM (*p* = 0.001, Figure 2B). Moreover, patients with the need for a perioperative RBC transfusion achieved a median OS of 2 months (95% CI 0.6–3.4), which was significantly reduced compared with the median OS of 13 months (95% CI 10.0–16.0) in patients without a perioperative RBC transfusion (*p* < 0.0001, Figure 2C). A total of 2 out of 19 patients (11%) with PBT exhibited severe intraoperative bleeding because of an intraoperative vascular injury. A further 9 patients (47%) suffered from highly vascularized metastatic lesions. Patients with the need for postoperative PMV achieved a median OS of < 1 month, which was also significantly inferior compared with the median OS of 12 months (95% CI 9.2–14.8) in patients without postoperative PMV after a surgical BM treatment (*p* < 0.0001, Figure 3A).

Furthermore, patients who exhibited PAE after the surgical treatment of BM achieved a median OS of 1 month (95% CI 0.0–3.1), which was significantly decreased compared with the median OS of 12 months (95% CI 9.1–14.9) in patients with an unremarkable postoperative course (*p* < 0.0001, Figure 3B). 

A total of 112 patients (72%) received postoperative adjuvant oncological therapy. Patients with postoperative chemotherapy, immunotherapy and/or radiotherapy exhibited a mOS of 20 months compared with 5 months for patients without a postoperative adjuvant treatment (*p* < 0.001).

Patients with a surgically treated infratentorial BM of NSCLC suffered from a significantly higher mortality rate after 12 months compared with patients with a supratentorial location of a surgically treated BM (70% vs. 45%, *p* = 0.004, OR 2.9). Furthermore, patients with multiple BM at the time of surgery also experienced a significantly higher mortality rate after 12 months compared with patients with singular BM (67% vs. 47%, *p* = 0.03, OR 2.3). Patients with the necessity of a perioperative transfusion of RBC suffered from a significantly higher mortality rate after 12 months compared with patients without a perioperative RBC transfusion (90% vs. 49%, *p* = 0.001, OR 8.9). All patients with a need for postoperative PMV for >48 h died within 12 months of surgery whereas 52% of patients without PMV were deceased within 12 months of surgery (*p* = 0.02, OR 14.0). Furthermore, patients suffering from PAE exhibited a significantly higher mortality rate after 12 months compared with patients with an uneventful postoperative course (85% vs. 51%, *p* = 0.022, OR 5.3).

### 3.5. Multivariate Analysis

**Figure 4:**
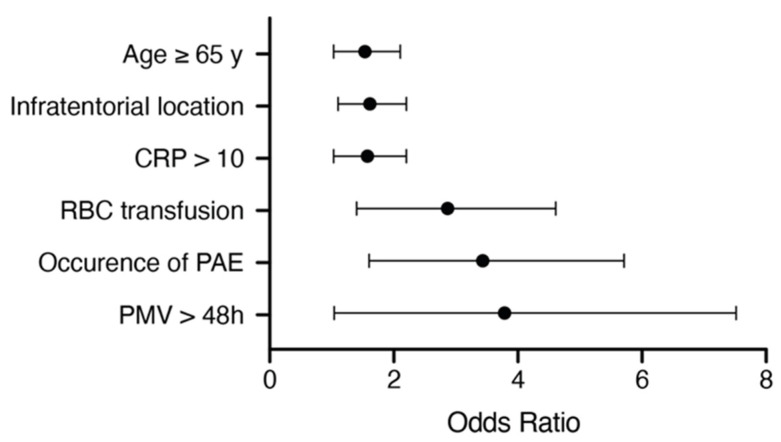
Results from the multivariate analysis. CRP: C-reactive protein; PAE: perioperative adverse events; PMV: prolonged mechanical ventilation; RBC: red blood cells.

We conducted an additional multivariate survival analysis using a proportional hazards regression analysis (Cox regression model) to identify the independent predictors of OS in patients with BM from NSCLC. The multivariate analysis confirmed the variables “age ≥ 65 years” (*p* = 0.036, OR 1.46, 95% CI 1.03–2.1), “infratentorial location of BM” (*p* = 0.023, OR 1.53, 95% CI 1.1–2.2), “preoperative CRP > 10 mg/L” (*p* = 0.034, OR 1.49, 95% CI 1.03–2.2), “perioperative RBC transfusion” (*p* = 0.001, OR 2.58, 95% CI 1.4–4.6), “postoperative PMV > 48 h” (*p* = 0.042, OR 2.79, 95% CI 1.04–7.5) and “occurrence of PAE” (*p* = 0.001, OR 2.98, 95% CI 1.6–5.7) as significant and independent predictors of a poor OS after a surgical treatment of BM seeded by NSCLC (Figure 4). 

## 4. Discussion

### 4.1. Clinical Admission Status

Neither the comorbidity burden based on an age-adjusted CCI nor the preoperative assessment according to the ASA classification were found to have a significant impact on the median survival of patients with BM in the present study. The lack of a significant association of comorbidities with survival in the present patient cohort does not support performing operations without any critical evaluation but it does indicate that with a careful selection and proper preoperative work-up and, if necessary, the improvement of certain clinical factors, the impact of comorbidities may be relegated to the background. In contrast, the preoperative KPS with a threshold value of 70 showed a significant influence on survival (*p* < 0.0001). The self-reliance assessed in the preoperative KPS represents a widely used and established survival prognosis instrument for patients with brain metastases as well as for patients suffering from a glioblastoma [11,17,18].

### 4.2. Tumor Burden and Location

The surgical treatment of patients with multiple brain metastases remains a controversial issue. In the present study, patients with symptomatic or space-occupying BM were treated surgically despite the presence of additional BM. Previous studies have shown a reversal or even a stabilization of neurological symptoms when single and/or multiple BM are surgically resected [14]. Modern neurosurgery also enables the resection of highly eloquent BM using appropriate intraoperative monitoring [15]. Thus, the preoperative, interdisciplinary evaluation raises the question whether a reduction of the tumor burden can or should be achieved despite multiple metastases and with what risk/benefit this is associated for the patient. Obviously, the presence of multiple intracranial metastases represents a highly advanced disease state, which often cannot be further controlled even by systemic therapy or high-precision radiation techniques. The reduced overall survival of patients with NSCLC and multiple BM in the present study demonstrates the need for a differentiated indication but also illustrates the feasibility of BM resection in multiple affected patients. 

The present study indicates no survival disadvantage in patients with BM located in the posterior fossa. This is concordant with other case studies in which a worsening of the prognosis related to the infratentorial localization in metastatic disease has been observed [16,17]. Most likely, the restricted amount of space, the proximity to highly eloquent brain regions and the risk of obstructive hydrocephalus (e.g., due to BM or radiation therapy) are among the most probable reasons [17]. 

### 4.3. C-Reactive Protein

Preoperative CRP > 10 mg/L has been identified as significant predictor for a poor overall survival. C-reactive protein is a representative acute phase reactant, with rapidly increasing levels in response to inflammation being one of the most commonly used systematic inflammation markers [18]. However, CRP may also increase in patients with malignancies without concomitant infections. Inflammatory reactions may also be triggered by tumor necrosis or tumor-related tissue damage, thereby releasing inflammatory factors. Previous studies have shown that increased CRP levels are associated with a reduced survival rate [18,19,20]. In particular, the increase in pretreatment CRP levels in serum has been identified as an independent prognostic factor for various types of solid tumors and NSCLC [20]. Although the underlying molecular mechanisms for this association remain unclear, a CRP increase appears also to be a sign of tumor proliferation and progressive involuntary weight and lean tissue loss, both of which are crucial elements in estimating cancer survival [20,21].

### 4.4. Postoperative Course

We suggest that a perioperative RBC transfusion is a significant predictor of poor survival after surgery for BM secondary to NSCLC. In the context of cancer, a transfusion-related immune modulation is often cited as the leading factor for poor oncological outcomes due to a suspected immunosuppressive effect of donor WBC in transfusions alongside aged erythrocytes and storage time [19,20,21]. In addition to the potential immunomodulatory aspects, the complexity and the potential blood loss of the operative procedure also have to be considered. In accordance with the results of the present study, the need for postoperative PMV was previously described as an important outcome parameter in patients with various types of cancer [22]. In addition to the lack of available capacities for re-convalescence and the complications of intensive care, the resulting delay of postoperative adjuvant (systemic) treatment is to be mentioned as a possible explanation for the exceptionally poor prognosis associated with PMV.

As is now shown in this study as well as previously, postoperative complications contribute significantly to an increased mortality after a surgical treatment of brain metastases [12]. A differentiated analysis of possible risk factors enables the identification of potential high-risk patients prior to surgery and thus leads to an extended surveillance or the implementation of an alternate treatment method (e.g., radiation therapy alone) with perhaps a more suitable risk/benefit ratio.

Along these lines, stereotactic radiosurgery has recently been reported to be associated with an improved local control of pretreated metastatic lesions compared with surgical resection [23]. It is therefore still to clarify whether the periprocedurally collectable prognostic variables in the course of the surgical treatment of BM in NSCLC patients will also pertain in the setting of stereotactic radiosurgery as a further oncosurgical treatment modality. Further studies will be needed in order to delineate the potential differences in the significance of the various prognostic parameters for both of these treatment modalities.

The present study identifies several laboratory and early clinical parameters that correlate to worsened survival rates in NSCLC patients with BM but are also known to indicate an impaired prognosis in non-metastatic lung cancer disease. However, especially for a treatment decision in advanced metastatic stages of cancer with the issue of further neurosurgical treatment, the identification of prognostic parameters such as the metastatic tumor burden, postoperative prolonged mechanical ventilation and elevated levels of perioperative RBC transfusions might contribute to an improved assessment of individual treatment benefits and treatment escalation in these late stages of NSCLC. Further studies might also assess the impact of several histopathological characteristics of the tumor of the primary site with regard to the survival outcomes in metastatic NSCLC. An elevated mitosis number objectified by the Ki-67 index is under debate as to whether it is associated with a poor prognosis [24]. Meta-analyses have found an inverse correlation between Ki-67 and survival [25,26] whereas other authors have raised doubts about these reports [27]. Further histopathological parameters such as elevated levels of tumor lymphocytic infiltration were found to be associated with improved survival in completely resected early NSCLC whereas vascular and lymphatic invasions indicate an early tumor recurrence and a worsened survival [28,29]. It has to be investigated whether these pathological characteristics might, in contrast to early non-metastatic NSCLC, also contribute to a more comprehensive estimation of survival in NSCLC patients in the course of an advanced metastatic stage of lung cancer disease.

### 4.5. Limitations

The present study has various limitations. The data collection was performed retrospectively. Patients were not randomized but treated according to the preferences of the treating physicians. Nevertheless, the present study on a comparatively large patient population investigates various aspects of surgically treated but interdisciplinarily managed patients with brain metastases and thus provides data from a collaborative research center on decision-making in everyday (neuro)oncological practice and also for the potential initiation of subsequent studies.

## 5. Conclusions

The present study identified several risk factors for a poor OS in patients that had undergone a surgical therapy of BM in the course of advanced NSCLC disease. In addition to patient age and infratentorial BM location, preoperative CRP, RBC transfusion, postoperative PMV and the development of postoperative complications were determined to negatively affect the survival after a surgical treatment of BM from NSCLC. These findings might guide a better risk/benefit assessment in the course of metastatic NSCLC therapy and might help to more sufficiently cope with the challenges of cancer therapy in these advanced stages of disease.

## Figures and Tables

**Table 1:** Patient characteristics.

Patient Characteristics	N of Patients (*n* = 154)
Median age at surgery	63 (range 40–86)
Female sex	74 (48%)
Median preoperative KPS	80 (range 30–100)
ASA ≥ 3	89 (58%)
Age-adjusted CCI > 10	79 (51%)
Multiple BM	54 (35%)
Infratentorial location of BM	54 (35%)
Admission CRP > 10 mg/dL	33 (21%)
Admission WBC > 12 G/L	93 (60%)
Postoperative PMV ≥ 48 h	7 (5%)
Perioperative RBC transfusion(within 5 days)	19 (12%)
Occurrence of PAE	13 (8%)
12 months of mortality	83 (54%)
Median OS (mo)	11 (95% CI 8.2–13.8)

ASA: American Society of Anesthesiologists; BM: brain metastases; CCI: Charlson comorbidity index; CRP: C-reactive protein; d: days; KPS: Karnofsky performance status; mo: months; PAE: perioperative adverse events; PMV: prolonged mechanical ventilation; RBC: red blood cells; WBC: white blood cells; yrs: years.

## Data Availability

The authors confirm that the data supporting the findings of this study are available within the article.
